# Flavonoid-Rich Extracts from Lemon and Orange By-Products: Microencapsulation and Application in Functional Cookies

**DOI:** 10.3390/foods14193346

**Published:** 2025-09-26

**Authors:** Giovanna Dellapina, Giovanna Poli, Vanna Moscatelli, Daniela Magalhães, Ana A. Vilas-Boas, Manuela Pintado

**Affiliations:** 1 Experimental Station for the Food Preserving Industry, Area of Transversal and Multidisciplinary Services—Consumer Science Division, Viale Tanara 31/a, I-43121 Parma, Italy; giovanna.dellapina@ssica.it (G.D.); giovanna.poli@ssica.it (G.P.); vanna.moscatelli@ssica.it (V.M.); 2 CBQF—Centro de Biotecnologia e Química Fina, Laboratório Associado, Escola Superior de Biotecnologia, Universidade Católica Portuguesa, Rua Diogo Botelho 1327, 4169-005 Porto, Portugal; dmagalhaes@ucp.pt (D.M.); avboas@ucp.pt (A.A.V.-B.)

**Keywords:** upcycled ingredient, polyphenols, circular economy, microencapsulation, sensory analysis, functional bakery products

## Abstract

Citrus by-products are increasingly recognized as a valuable source of bioactive compounds (BCs), particularly flavonoids. Their incorporation into food matrices as functional ingredients aligns with sustainability goals and consumer demand for health-promoting products. However, challenges such as poor stability and undesirable sensory properties limit their direct use in food systems. This study aimed to develop and evaluate functional cookies enriched with microencapsulated flavonoid-rich extracts derived from lemon and orange peels. Flavonoids were extracted with hydroethanolic solvent and characterized by HPLC-DAD. The extracts exhibited high total flavonoid contents: 1960.1 mg/L for orange and 845.7 mg/L for lemon. The extracts were encapsulated using a 1% sodium alginate and 1.36% corn starch blend, producing thermally stable microbeads with flavonoid retention higher than 85% after heating at 230 °C for 30 min. These microbeads were incorporated into gluten-free oat and buckwheat cookies, delivering 166.11 mg/100 g (orange) and 177.13 mg/100 g (lemon) of flavonoids in the product, which covers approximately one-third of the recommended daily intake. Sensory analysis using triangle tests (ISO 4120) (n = 23) showed no significant difference (*p* > 0.05) between control and enriched cookies, indicating successful masking of potential bitterness or astringency associated with flavonoids. These results demonstrate the effectiveness of microencapsulation in protecting citrus flavonoid-rich extracts and support the development of sustainable, health-oriented bakery products. Moreover, this approach promotes the valorization of agro-industrial by-products, contributing to a more circular food supply chain.

## 1. Introduction

Citrus by-products represent a rich and underutilized source of high-value bioactive compounds (BCs), particularly phenolic compounds, offering great potential for food innovation and feedstock valorization [1,2]. Their reuse contributes directly to several Sustainable Development Goals, including SDG 3 (good health and well-being), SDG 12 (responsible consumption and production), and SDG 13 (climate action). Therefore, efforts are being made to increase the added value of food by-products through the production of functional ingredients, thereby increasing the income derived from these by-products, such as the extraction of specific BCs. Citrus fruit is a significant source of flavonoids in the human diet, such as hesperidin, eriocitrin, nobiletin, and phenolic acids, including ferulic acid, caffeic acid, and p-coumaric acid [3]. The consumption of these polyphenols has been associated with multiple health-promoting bioactivities, including antioxidant, anti-inflammatory, anticancer, antimicrobial, antiallergic effects, cardioprotective, and antidiabetic, as well as gut microbiota modulation [4,5,6]. They contribute to the prevention of chronic diseases and support long-term health [7,8]. Studies show that polyphenol-rich diets (>650 mg/day) are linked to lower mortality, while lower intakes <500 mg/day show higher mortality risk [9]. However, excessive intake (>1000 mg/day) from supplements may raise safety concerns related to toxicity. Nevertheless, estimating actual intake remains challenging due to the limited compositional data available for processed foods.

**Figure 1:**
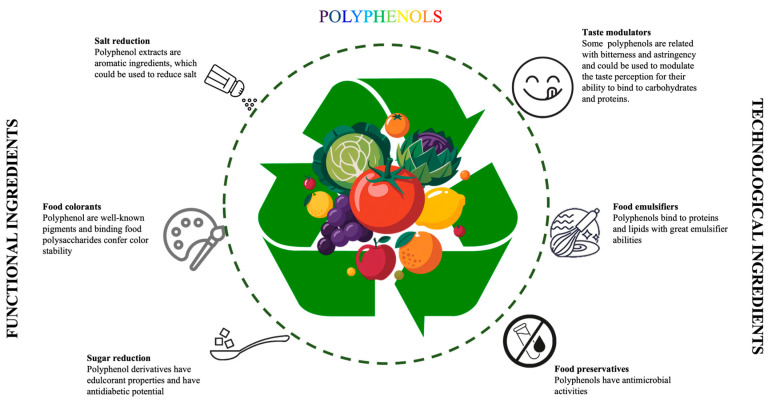
The potential effects of polyphenols in the food industry.

In addition to the health-promoting benefits, polyphenols offer a promising path for food improvement due to their functional and technological properties (Figure 1). Several studies have highlighted that polyphenols are promising natural alternatives to synthetic additives, thanks to their inherent antioxidant and antimicrobial properties. These BCs contribute to product stability by neutralizing free radicals, which helps preserve freshness, color and flavor [10,11]. Additionally, their antibacterial activity inhibits the growth of spoilage and pathogenic microorganisms, thereby extending the shelf-life of food products [12].

Despite their recognized antioxidant potential, polyphenols are highly susceptible to oxidative degradation and sensitive to environmental factors such as heat, light, moisture, and oxygen, which can compromise their stability and bioactivity [13]. Therefore, protecting these compounds from environmental factors, such as heat, moisture, and oxygen, as well as from degradation in the gastrointestinal tract, is essential. Moreover, the sensory characteristics of liquid extracts, such as bitterness and astringency, can decrease consumer acceptability, emphasizing the need for effective masking strategies [14]. Astringency is a trigeminal sensation perceived as dryness, roughness, and tingling in the mouth, often caused by tannins and organic acids. These compounds interact with salivary proteins, especially proline-rich proteins, leading to precipitation, and may also activate G-protein-coupled receptors or bind to oral epithelial cells. Bitterness, on the other hand, arises from the activation of specific bitter taste receptors (TAS2Rs), of which humans possess 25 TAS2Rs that enable the detection of a wide range of bitter-tasting molecules with diverse structural properties [15]. Many polyphenols act as TAS2R agonists, even at low concentrations, which may trigger negative taste responses and compromise the acceptability of functional foods [16].

Microencapsulation has emerged as an effective strategy to improve the stability, sensory masking, and handling of BCs, particularly polyphenols. It enables the transformation of liquid extracts into dry, stable, and easy-to-handle powders for food applications and storage at room temperature [17]. The literature data show that the encapsulation process provides the formation of a barrier that may interfere with parameters such as temperature, light, oxygen, and moisture, and maintain the stability of the encapsulated component. In this way, it is possible to preserve the taste and aroma, mask the bad taste and odor and increase the stability and bioavailability of sensitive substances. Mostly, alginates are used as a coating material for encapsulation by ionic gelation. Alginate is accepted as a coating material by the FDA (Food and Drug Administration) and the EFSA (European Food Safety Authority). The most important advantages are that the product does not adversely affect flavor during consumption and is resistant to both thermal and chemical processes [18]. To test the ability of microencapsulation to prevent thermal degradation, Flamminii et al. [19] conducted thermal analyses on freeze-dried microbeads, which revealed increased thermal stability of encapsulated polyphenols.

Ionic gelation is the most widely used method for microencapsulation in the food sector due to its simplicity, mild processing conditions, and use of food-grade biopolymers. In this method, BCs are mixed with a carrier material, commonly sodium alginate, which is a food-grade polysaccharide widely recognized for its gelling ability. When an aqueous solution of sodium alginate is introduced into a calcium chloride solution, the divalent calcium ions interact with the guluronate blocks of the alginate chains, inducing gelation and forming spherical hydrogel beads with uniform size and structure. These alginate beads can entrap sensitive compounds, protecting them from environmental and processing-related stress. However, both sodium alginate and phenolic compounds are hydrophilic, which may reduce encapsulation efficiency and limit the control of release profiles [20]. To improve performance, other polymers such as starch, chitosan, or polyvinyl alcohol have been studied for blending with alginate to modify the bead structure and enhance interaction with the BCs [21]. The combination of alginate with starch as carrier, reinforce bead structure and improve interactions with the BCs, thus enhancing encapsulation efficiency and stability. In addition, potentially masking the gluten-free baked products represents a promising and underexplored strategy to enhance the practical application of citrus extracts in functional foods.

In parallel, consumer demand for “healthy,” “natural,” and “sustainable” products is reshaping food consumption patterns and driving innovation in the food industry. There is growing interest in functional foods that reduce synthetic additives and offer health benefits beyond basic nutrition. This shift is enhanced not only by increased awareness and affluence but also by the rising prevalence of food- and lifestyle-related diseases, such as obesity, diabetes, and allergies. Consumers are increasingly attentive to the origin, processing, and composition of food products, favoring options that support long-term health, healthy aging, and environmental sustainability [22]. Agro-industrial by-products, due to their rich composition and functional potential, represent valuable resources for food innovation. Their incorporation into functional or nutraceutical products aligns with circular economy principles, promoting sustainability, reducing food waste, and contributing to zero-waste strategies [23].

The development of functional foods requires a clear understanding of the number of bioactive compounds needed to achieve well-being. Equally important is compliance with current regulations governing food additives, such as Regulation (EC) No. 1333/2008 of the European Parliament and the Council of the European Union (2008) [24]. Nevertheless, many of these functional molecules are characterized by high reactivity and poor chemical stability, which necessitates their use in encapsulated forms. Although these compounds are known for their health-promoting properties, their addition into food products may unpleasantly affect sensory attributes, often resulting in undesirable bitterness or astringency that can lead to consumer rejection.

This study aimed to incorporate flavonoid-rich extracts from lemon and orange by-products into microbeads through ionic gelation and evaluate their application in gluten-free functional cookies with at least one-third of the recommended daily intake of polyphenols per serving. The extracts were encapsulated to enhance stability, protect their bioactivity against thermal degradation and improve sensory properties masking possible unpleasant taste and/or smell. The retention of flavonoids after baking and sensory acceptability were assessed to validate their potential as an innovative and functional product based on circular economy concepts.

## 2. Methodology

### 2.1. Plant Materials (Lemon and Orange By-Products)

Lemon by-products (Portuguese *Eureka* variety), consisting of peels and pulps, were purchased from a supermarket in May 2022, supplied by the Frusoal company, located in the Algarve region of Portugal. The fresh lemon by-products were subjected to essential oil extraction in a Clevenger apparatus at 100 °C for 2 h. The remaining by-products were then frozen at −18 °C for a maximum of 1 week until further use for phenolic compound extraction.

Orange by-products (Lane Late cultivar, PGI Algarve Citrus) were collected in May 2022 from juice squeezer machines (Speed S, Zumex^®^, Valencia, Spain) operating in Continente^®^ store (Matosinhos, Oporto, Portugal). These by-products consisted mainly of peels with some residual pulps. After collection, they were transported under cold chain conditions (within 2 h) and subjected to essential oil extraction by hydrodistillation in a stainless steel pilot-scale still (ESAC-IPC, Coimbra, Portugal). The remaining peels were then frozen at −18 °C for a maximum of 1 week until further use for phenolic compound extraction.

### 2.2. Lemon and Orange Flavonoids-Rich Extracts Development

Fresh ground lemon and orange by-products (in separated) were subjected to a hydroethanolic extraction using a 1:9 (*m*/*v*) solid-to-solvent ratio with 60% (*v*/*v*) ethanol. The extraction was carried out under controlled magnetic stirring at 200 rpm, using 300 g of sample, at room temperature (25–27 °C) for 50 min. The mixture was then centrifuged at 5000× *g* for 10 min at 4 °C using a ROTINA 420/420R (Hettich^®^, Tuttlingen, Germany) centrifuge. The supernatant was collected and concentrated sixfold by reverse osmosis (Pilot plant developed by ORM (Belas, Portugal) equipped with 2.5 S seawater pressure vessel and a 1 m^2^ Filmtec membrane SW302540 (Dow Chemical Company, Midland, TX, USA). Subsequently, the ethanol fraction was evaporated at 40 °C under 175 mbar pressure using a rotary evaporator R-210 (BÜCHI, Flawil, Switzerland), yielding an aqueous liquid extract used for further analysis.

### 2.3. Characterization of Flavonoids-Rich Extracts by HPLC-DAD

The phenolic compounds in the citrus extracts were identified and quantified using a Waters Alliance e2695 separation module system interfaced with a photodiode array UV/Vis detector 2998 (PDA 190–600 nm; Waters, Mildford, MA, USA). The separation occurred in a reversed-phase C18 column (Avantor^®^ Alltima HP, C18-AQ (100 A°; 5 μm; 4.60 mm; 250 mm), Radnor, PA, USA) at 25 °C. The mobile phase, gradient elution, and data acquisition and analysis were prepared according to [25]. Briefly, the mobile phase was composed of solvent A: water/acetonitrile/TFA (94.9/5/0.1%) and solvent B: acetonitrile/TFA (99.9/0.1%) with the elution gradient: 0–1 min 0% B; 1–30 min 21% B; 30–42 min 27% B; 45–55 min 58% B; 55–60 min 0% B, and kept another 1 min at 0% B. Flow rate was 1 mL/min, the oven temperature was set as 25 °C, and the injection volume was 20 µL. Data acquisition and analysis were performed using Empower 3 Chromatography Data software (Build 3471, Database Version 7.20.00.00, Waters Corporation, Milford, MA, USA). Detection was performed at 280, 320, and 350 nm, and phenolic compound identification was achieved by comparing the retention times and absorbance spectra with those of pure standards. Three independent analyses were performed, and the results were expressed as milligrams per liter of dry extract (mg/L DE).

### 2.4. Total Flavonoids Quantification (Spectrophotometric Method)

Total flavonoids were measured using the aluminum chloride assay [26], with some modifications. A known volume of extract was placed in a 10 mL volumetric flask. Distilled water was added to bring the total to 5 mL, followed by the addition of 0.3 mL NaNO_2_ (1:20) (Carlo Erba Reagents, Val de Reuil, France). Five min later, 3 mL AlCl_3_ (1:10) (Sigma Aldrich, St. Louis, MO, USA) was added. After an additional 6 min, 2 mL of 1 M NaOH (Carlo Erba Reagents, Val de Reuil, France) was added, and the total volume was adjusted to 10 mL with distilled water. The solution was thoroughly mixed, and the absorbance was measured against a blank sample in a Shimadzu spectrophotometer (UV1601-Shimadzu, Duisburg, Germany). Quercetin (USP reference standard, Sigma-Aldrich, St. Louis, MO, USA) served as the standard for calculating the calibration curve. Samples were read at 410 nm, and the results are expressed in milligrams of quercetin equivalents per 1000 g of product. The flavonoid content was calculated using the following linear equation based on the calibration curve: y = 805.63x − 6.5079, R^2^ = 0.9993, where x represents the absorbance, and y denotes the flavonoid content in mg kg^−1^. Analyses for the determination of total flavonoids were conducted in triplicate.

### 2.5. Microbeads: Development and Characterization

To identify the encapsulation carrier material that allowed to achieve beads with (a) better efficiency to retain the extract during gelling in an aqueous environment, and (b) low tendency to pack during the storage period, several blends have been tested:

- Na alginate solution (0.5, 0.8, 1.0, 1.2 and 1.5%) (B&V srl, Gattatico, RE, Italy);
- Na alginate solution with PVA (polyvinilalcol, A.C.E.F. srl, Fiorenzuola d’Arda, PC, Italy) (0.5, 0.8 and 1%) [27];
- Na alginate solution with corn food starch (Maizena, Pancalieri, TO, Italy) (1.2, 1.36, 2.0%) [28];

The different solutions under study were passed through a Büchi Encapsulator B390 (BÜCHI Labortechnik AG, Flawill, CH), a laboratory-scale instrument capable of producing microcapsules via extrusion and ionic gelation. In this technique, the solution to be encapsulated flows through one or two nozzles, forming a laminar liquid jet which is divided into micro-drops due to the action of superimposed vibrations. The frequency of the vibration determines the quantity of drops produced per second. To separate the micro-drops formed, an electrical charge is induced onto the surface of the droplets, which, falling into a suitable solution, solidify. The size of the micro-drops produced depends on the section of the nozzle chosen. Furthermore, by adjusting factors such as temperature (in our conditions at 45 °C) and pressure, the operating conditions are optimized to obtain homogeneous beads. The nozzle diameter varies from 80 to 1000 μm, and the microcapsule sizes range from 120 μm up to 2.4 mm in diameter (Table 1). Table 1 displays the parameter ranges for conducting tests. It indicates that each process can vary due to different factors; for example, flow is controlled by pressure modulation, which is also affected by the fluid’s viscosity and the operating and ambient temperatures. Therefore, these are guide values that may fluctuate even within the same microencapsulation process.

The gelling solution consisted of calcium chloride 0.2 M (A.C.E.F. srl, Fiorenzuola d’Arda, PC, Italy). In addition, to extend the stability of beads, the gelling capacity of chitosan from mushrooms (Bonding Chemical, Caty, TX, USA) was evaluated by adding 0.5% to the calcium chloride solution [29,30]. The tests that determined the choice of carrier material and microencapsulation process conditions were conducted with lemon extracts. The method efficiency evaluation was based not only on the percentage ratio between encapsulated BCs and added BCs, but also on factors such as particle sphericity, avoiding collapse phenomena, low sticking tendency during drying, size uniformity, and ability to retain the active ingredients during drying.

The orange and lemon extract beads obtained with the optimal method based on encapsulation efficiency were dried in a thermostatic chamber (Shaker Incubator Argolab SKI4, Carpi, MO, Italy) in a thin layer at 40 °C for 12 h. This prevents the thermal degradation of BCs [31], and the dehydration level achieved (with an average weight loss of 95–97%) also inhibits microbial growth during storage. The dried microbeads were then cooled at room temperature and vacuum packed (Vacuum Packaging Machine—Model MIDY, Parma, Italy) in flexible bags (Undivac 140—Cryovac, Elmwood Park, NJ, USA) designed for resistance from O_2_ and humidity. The packed beads were then stored in a laboratory cabinet in the dark at room temperature and used within a week. Dried microbeads were then analyzed for their total flavonoid content. Further, 0.8 g of dried beads rehydrated with 15 mL of water were crushed for 5 min with a Coors™ porcelain pestle in a Coors™ porcelain mortar (Sigma-Aldrich, St. Louis, MO, USA). The resulting liquid fraction was transferred to a paper filter (Whatman^®^ prepleated qualitative filter paper for technical use, Merck KGaA, Darmstadt, Germany). The residual solid fraction in the mortar was subjected to three more consecutive extractions using alternately 15 mL of 96% ethanol (VWR Chemicals, Fontenay-sous-Bois, France) and 15 mL of distilled water. The extracts (60 mL) from the subsequent filtrations were then mixed and analyzed.

#### Production Cost Estimation

The estimation of microbead production costs was based on the calculation of chemical and energy expenses plus overheads, which are considered 80% of the chemical and energy costs, including waste management, equipment depreciation, labor costs, and other expenses [32]. In addition, the costs of flavonoid-rich extract were calculated, as it is the most representative part of the microbeads. The key assumptions used to estimate the production process costs are summarized in Table 2, considering a target output of 450 mL of flavonoid-rich extract and 27 g dried beads. Energy requirements were based on the operating conditions of the laboratory-scale equipment used, while reagent costs were calculated using market prices for high-purity laboratory-grade chemicals. Capital expenditure (CAPEX) was not included in this estimation.

### 2.6. Development of Functional Cookies with Flavonoids-Rich Extracts

Gluten-free and nut-free cookie formulations were developed to ensure suitability for individuals with gluten intolerance or nut allergies. Buckwheat flour and oat-based ingredients were selected as the main components of the gluten-free cookie dough matrix Table 3. All ingredients were mixed with water in a KitchenAid mixer until a homogeneous dough was obtained. The dough was then flattened to approximately 0.5 cm thickness and cut into portions with a final weight of approximately 10.5 g each.

The microencapsulated flavonoids-rich extracts derived from orange (Orange Polyphenols Cookie—OPC) and from lemon (Lemon Polyphenols Cookie—LPC) were incorporated into the base cookie formulation (Control Cookie—CC). The amount of microcapsules added was calculated to provide approximately one-third of the recommended daily intake of polyphenols (≥500 mg/day), as suggested by Williamson and Holst (2008) [8]. This corresponded to 14 g of dried beads per 100 g of final dry product. All cookie samples were baked in an Electrolux air-O-steam IMQGS oven (MARAN Projekt GmbH, Hamburg, Germany) at 180 °C for 17 min. After baking, cookies were analyzed for their total flavonoid content. For flavonoid extraction, 25 g of cookies were crushed with a Coors™ porcelain pestle in a Coors™ porcelain mortar (Sigma-Aldrich, St. Louis, MO, USA). The resulting powder was rehydrated with 60 mL of distilled water 50: ethanol 50 solution and crushed again for 10 min, and transferred on a paper filter (Whatman^®^ prepleated qualitative filter paper for technical use, Merck KGaA, Darmstadt, Germany) and analyzed.

### 2.7. Sensory Analysis—Triangular Test

To assess the contribution of citrus flavonoid extracts to consumer acceptability, a comparison was made between cookies without microcapsules and those enriched with OPC and LPC extracts. This discriminatory method is used to determine differences or non-differences between two products based on one or more sensory attributes. The standard ISO 4120:2021 was applied [34]. Triangle tests were conducted to determine if there were statistically significant differences between the products in the two types. The conditions used to perform the tests set for difference were: α = 0.05, β = 0.20, pd = 40%, where α: is the probability of concluding that a perceptible difference exists when it does not (also known as type I error or significance level), β is the probability of concluding that no perceptible difference exists when at least one exists (also known as type II error) and pd is the proportion of the entire population of assessors able to distinguish between the two products.

A panel composed of 23 subjects, trained to perform this analysis, was used to conduct the test according to the standard ISO 4120:2021. Under the conditions adopted, the minimum number of correct answers required for statistical significance of the test was 12. To hide any possible color difference and focus the judges’ attention only on texture and/or taste-related aspects, red lights were used during evaluation sessions. An evaluation session was repeated under white solar light. The tests were conducted in single evaluation booths under constant and controlled environmental conditions (temperature and humidity), using dedicated facilities and equipment at the SSICA Sensory Analysis Lab, built and equipped according to the UNI EN ISO 8589:2014 standard and accredited for the execution of the main sensory tests [35] (Accredia Certificate n° 0122).

The triangle test involves presenting each subject with a triad consisting of two identical samples and one different sample. The assessor is then asked to taste the products from left to right and try to identify the odd sample. The booths assigned to the assessors are prepared according to a randomized block pattern to use an equal number of the six possible combinations of the two products compared. Samples were coded with a three-digit number identifying the product and presented to each judge. The coding of the samples is randomized, as is the order of presentation to the subjects. The subjects mustn’t be led to identify the samples based on their presentation (same quantity, appearance, size, shape). Data collection was conducted via terminals located in test booths, using dedicated software (Fizz, Biosystème, Couternon, France) that recorded the subjects’ responses directly.

### 2.8. Statistical Analysis

The data were presented as mean ± standard deviation of at least three independent experiments (n = 3). The statistical differences were investigated using a one-way ANOVA test with Statgraphics Plus 2.1 software (Statgraphics Technologies, Inc., The Plains, VA, USA), at a 95% confidence level, followed by Tukey’s test to control for multiple pairwise comparisons at an alpha level of 0.05.

## 3. Results and Discussion

### 3.1. Flavonoids-Rich Extraction

The identification and quantification of phenolic compounds by HPLC-DAD in orange and lemon extracts are presented in Table 4. In contrast, the total flavonoid content (TFC) of these extracts is outlined in Table 5. These citrus extracts contain a significant amount of flavonoids, with orange exhibiting the highest total content (1960.1 mg/L), compared to lemon extract (845.7 mg/L). These results are consistent with peer-reviewed studies reporting that orange peels generally contain higher levels of phenolic compounds and exhibit greater antioxidant activity compared to lemon peels [36,37]. In a recent solvent extraction study, the highest total phenolic content (TPC) in orange peel extracts using ethanol reached 387.7 mg gallic acid equivalent (GAE)/g, while lemon peel extracts reached only 238.1 mg GAE/g with the same solvent, showing orange peels have a significantly higher TPC [38]. Furthermore, a comparative study on fresh peels found that the total phenolic content of orange peel (158.54 mg GAE/100 g) was notably higher than that of lemon peel (114.58 mg GAE/100 g), and this trend remained consistent after different drying processes, emphasizing the predominance of phenolic compounds in orange peels [39]. In addition, the major phenolic compound identified and quantified by HPLC-DAD in orange extract was hesperidin (454.00 mg/L), followed by nobiletin (235.57 mg/L) and neoeriocitrin (223.88 mg/L). In lemon extract, the major phenolics identified were eriocitrin (357.30 mg/L) and hesperidin (100.98 mg/L). These results are consistent with previous review articles describing hesperidin and polymethoxylated flavones, such as nobiletin, as major constituents of orange peel, whereas eriocitrin is typically the predominant flavonoid in lemon peel [40,41]. This is also in line with the study of Mateus et al. [42], who reported hesperidin as the most abundant flavonoid in orange (2453.92 μg/g), while eriocitrin was the major phenolic in lemon (1249.87 μg/g). However, the direct comparison of values represents a limitation, since the studies report results in different units, making it difficult for readers to compare the data directly. Nevertheless, a qualitative comparison clearly supports the evidence of our conclusions.

The functional relevance of the main compounds identified here is twofold. From a health perspective, hesperidin is widely associated with antioxidant, vasoprotective and anti-inflammatory activities; nobiletin, a polymethoxylated flavone abundant in citrus peels, shows anti-inflammatory, neuroprotective and favorable lipid/glucose-modulating effects; and eriocitrin, the predominant lemon flavonoid, exhibits strong radical-scavenging capacity and antidiabetic potential [40]. Technologically, these flavonoids can enhance oxidative stability in fat-containing bakery matrices and thus contribute to product quality and shelf-life—particularly relevant for thermally processed goods, where compound stability is critical [43,44]. In line with this, Imeneo et al. [45] demonstrated that biscuits enriched with lemon pomace (rich in eriocitrin and hesperidin) had a higher phenolic content and antioxidant activity, and displayed a longer induction period to lipid oxidation than the controls, while maintaining acceptable sensory quality. Likewise, de Castro et al. [46] showed that orange juice by-product flour, characterized by high phenolic content and antioxidant potential, could be incorporated into cookies without detrimental effects on technological or sensory properties. Together, these findings support the health-oriented value of citrus flavonoids and their practical technological advantages when incorporated into bakery products.

### 3.2. Micro-Encapsulation Tests

**Figure 2:**
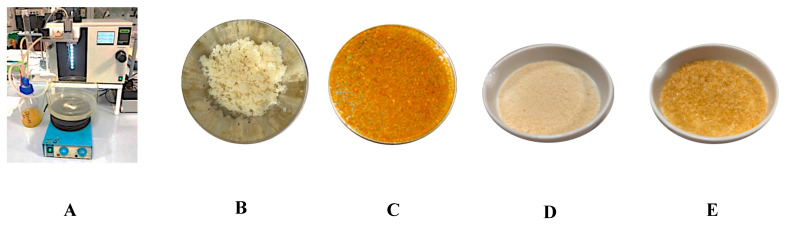
Micro-encapsulation of citrus extracts: (**A**) Micro-encapsulator; (**B**) Fresh lemon beads; (**C**) Fresh orange beads; (**D**) Dried lemon microbeads; (**E**) Dried orange microbeads.

The initial tests enabled the determination of the optimal carrier material for producing microcapsules to be inserted into bakery products (Figure 2). To increase the blend fluidity, the solution and micro-encapsulator were kept at a temperature of 45 °C. Below are the mixtures studied and the reasons for the carrier material choice:

- Beads of Na-alginate: Sodium alginate at 1% resulted in the most efficient formulation. Concentrations of 0.5–0.8% produced beads that were too soft and showed lower compound retention, while concentrations higher than 1% did not flow well through the nozzles and formed beads that were too hard. Confirming these observations, data from the literature [47] state that for a low alginate concentration (<1.0%), the viscous and surface tension forces are lower than the minimum ones required to counteract the effect of impact and drag, and almost no spherical particles are formed, probably due to the lack of enough carboxyl groups for gelation. This probably leads to higher diffusion rates from the beads to the external media.
- Beads of Na-alginate with starch: The beads produced with 1.36% corn starch had the desired spherical shape and retained the compounds more effectively.
- Beads with PVA (0.5, 0.8, and 1%): These beads collapsed to varying degrees, especially upon dehydration.
- Beads of alginate with chitosan: In this case, chitosan was added to the gelling bath (CaCl_2_ 0.2 M + 0.5% chitosan). These beads exhibited the highest retention of active substances during gelation; however, upon recovery from the gelling solution, they lost their spherical shape and tended to stick together.

Regarding bead dimensions, different nozzles were tested. The smaller beads (80–200 µm) compacted after drying, while the larger ones (300–750 μm) retained a grainy texture, with granulometry between that of sugar and flour. The 750 µm nozzle was selected. The 1% sodium alginate and 1.36% starch blend were identified as the most effective with efficiency varying between 82 and 85.5%. It enables (a) minimizing the loss of functional substances during the bead gelling process, (b) producing homogeneous beads, and (c) drying the beads for later use as food ingredients. Finally, the beads obtained using orange and lemon extracts with a 1% sodium alginate and 1.36% starch blend, and a 750 μm nozzle, were dried until a 97% weight loss was achieved.

**Figure 3:**
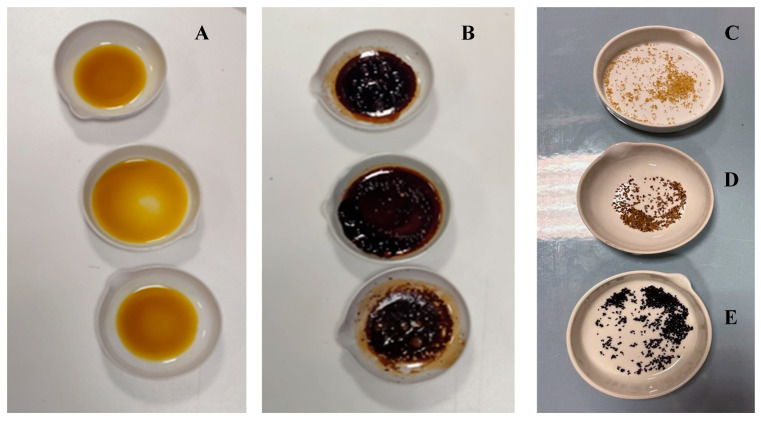
Heat treatments of extracts and dried beads: (**A**) Extracts before heat treatments; (**B**) Extracts after heat treatments at 180 °C; (**C**) Dried beads before heat treatments; (**D**) Dried beads after heat treatments at 180 °C; (**E**) Dried beads after heat treatments at 230 °C.

After drying, the beads were analyzed for total flavonoid content (Table 6). During the microencapsulation, it was observed that the phenolic compounds partially diffused into the gelling liquid (aqueous solution of CaCl_2_). Nevertheless, the analysis conducted on the dried beads revealed their suitability for use as a functional ingredient. To evaluate the protective effect of the microencapsulation process against the heat treatment to which the cookies were subjected, thermic treatments simulating the cooking process on pure lemon extract and dried lemon beads (30 min at 180 °C and 30 min at 230 °C) were conducted to check their suitability in baked products and any increase in heat resistance provided by encapsulation (Figure 3).

Tests indicate that after 30 min of heat treatment at 180 °C, the flavonoid content of lemon extract decreased by an average of 47.01% (*p* = 0.0004), while no statistically significant losses were observed in dried lemon beads (*p* = 0.1996) heat-treated up to 230 °C.

The results obtained for the flavonoid content in orange extracts after 30 min of heat treatment at 180 °C showed a statistically significant decrease of 47.23% (*p* = 0.0007). Furthermore, the significance letters shown in Table 6 indicate that a statistically significant difference was found between the flavonoid content in microcapsules before heat treatment and that of those heated for 30 min at 230 °C. Naturally, the color of the beads has undergone notable browning, which must be taken into consideration when using them as ingredients.

#### Production Cost Estimation

The estimation of production costs for the flavonoid-rich extracts and the corresponding microbeads is presented in Table 7. These values represent approximate laboratory-scale calculations and should be interpreted as indicative estimates rather than absolute production costs. The total cost of producing 450 mL of liquid-concentrated, flavonoid-rich extract was calculated at 42.16 €/kg, while the microbead production stage contributed an additional 5.60 €/kg, resulting in an overall cost of approximately 47.73 €/kg product.

The cost breakdown reveals that ethanol was the primary contributor during the extraction stage, accounting for 54% of the total extraction costs. This was followed by overheads (44%), which were empirically defined as 80% of the sum of chemical and energy costs to approximate labor, waste management, and depreciation. Energy consumption during stirring, centrifugation, and reverse osmosis represented a comparatively minor fraction. In contrast, the microencapsulation step was less costly overall, with energy use by the encapsulator and drying incubator, as well as overheads, being the main contributors (80%). These indicate that the extraction process is the cost-limiting step under the current conditions.

Compared to techno-economic analyses reported for the recovery of flavonoids and hesperidin from orange peel, our laboratory-scale estimate is substantially higher. Restrepo-Serna and Cardona-Alzate (2024) [48] reported that solvent extraction with 50% ethanol achieved the best economic performance, with a positive net present value and a profit margin of 45%. Similarly, Ortiz-Sanchez et al. (2021) [49] showed that integral biorefineries using orange peel to obtain pectin, essential oil, and polyphenols could only reach feasibility at larger scales, with costs strongly influenced by solvent use and energy integration. In both cases, the main cost drivers were solvents and utilities, in agreement with our results, where ethanol dominates the balance. Additionally, other studies have reported extremely high production costs for phenolic-rich extracts, even when different plant matrices were employed. Adeyi et al. (2022) [50] estimated a unit production cost of ≈487 €/kg for phenolic extracts from papaya leaves using heat-assisted extraction. Their analysis identified solvents (particularly ethanol), energy, ultrapure water, and overheads as the most significant contributors, which is consistent with the cost structure observed in our study. This further confirms that the use of laboratory-grade reagents and utilities, combined with overhead assumptions, can markedly inflate costs at small scale. This is mainly due to the reliance on laboratory-grade solvents and reagents, which are substantially more expensive than food-grade or industrial alternatives. In addition, no solvent recovery was considered here, whereas industrial processes typically include efficient ethanol recovery systems that can significantly reduce consumption and associated costs. Furthermore, laboratory-scale equipment is prone to lower yields and higher losses, which artificially inflate unit costs compared to scaled-up operations.

These considerations emphasize that the values reported in this study must be interpreted as an upper-limit scenario. In a realistic industrial context, the adoption less expensive solvent for extraction, the solvent recovery, and optimized energy management would substantially lower costs. Nevertheless, the estimated figures suggest that incorporating microbeads into low-cost bakery products, such as cookies, may face economic constraints. More promising applications may lie in premium functional foods, nutraceuticals, or clean-label formulations, where consumers are willing to pay a higher price for added health benefits and sustainability credentials.

Overall, this preliminary analysis offers valuable insights into the cost drivers of the process and underscores the importance of techno-economic assessments as a complementary tool to laboratory research. Future studies should integrate pilot-scale data, evaluate process scalability, and conduct sensitivity analyses on key variables such as solvent price, recovery efficiency, and bead yield to establish the true cost-effectiveness of flavonoid-enriched microbeads in food applications.

### 3.3. Flavonoids-Enriched Cookies Production and Evaluation

**Figure 4:**
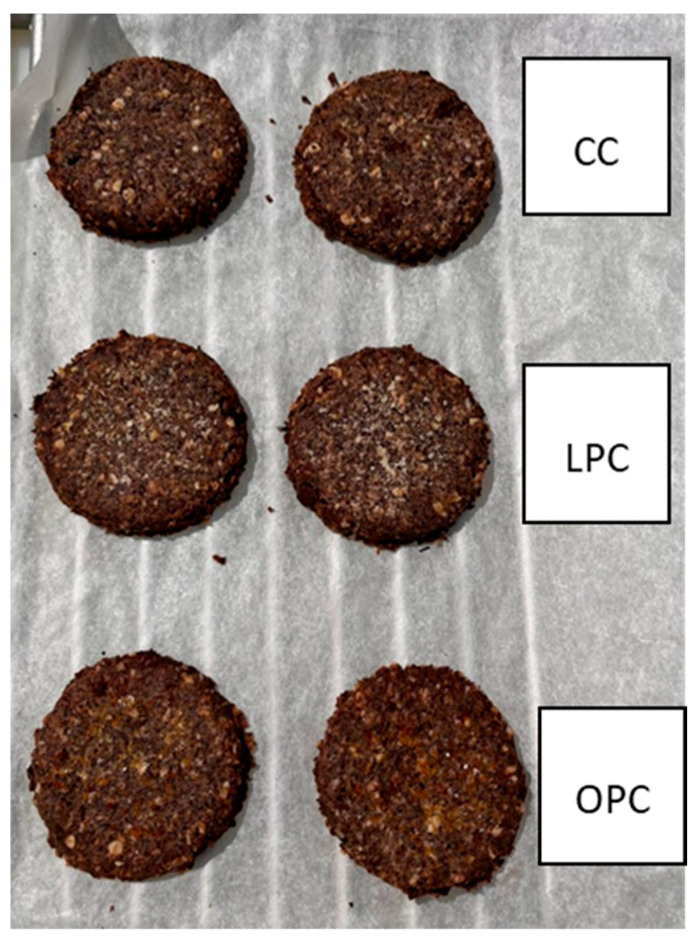
Flavonoid-enriched cookies: Control Cookie (CC), Orange Polyphenols Cookie (OPC) and Lemon Polyphenols Cookie (LPC).

The cookies were analyzed to verify that, following the baking process, the technological process used was able to preserve the required flavonoid content. The dark color of the cookies was due to the presence of chocolate in the dough. At the same time, no visually apparent differences were noted between the various formulations in terms of the presence of orange or lemon beads (Figure 4). The three different types of cookie were evaluated in triplicate for their total flavonoid content (Table 8). The amount determined per 100 g of cookies (approximately 6 units) fully satisfies one-third of the daily dietary requirement. The results confirm that microencapsulation effectively preserves the flavonoid content from thermal degradation during the cooking process.

### 3.4. Sensory Analysis

Furthermore, the three cookies types were analyzed by means of sensory comparison tests to investigate whether consumers could perceive the addition. The cookies OPC and LCP were compared with the Control (CC) using the triangle test (ISO 4120:2021). Two triangle tests were conducted: OPC vs. CC and LPC vs. CC. As stated, under the conditions adopted, the minimum number of correct answers required for statistical significance of the test was 12. In the triangular test of cookies enriched with orange extract, 6 out of 23 assessors (26%) identified the different sample within the triad of samples under red light conditions while 5 out of 23 under solar light. In contrast, in the evaluation of cookies with lemon extract beads, only 3 out of 23 assessors identified the different sample under both lighting conditions.

The triangle test is a forced-choice procedure. Assessors are not allowed the option of reporting “no difference”. An assessor who detects no difference among samples should be instructed to select one randomly and to indicate that the selection was only a guess in the scoresheet. The tables provided by the ISO standard for interpreting results consider the possibility that one-third of the judges may identify the different samples randomly. For both cookie formulations, the number of correct responses was below the minimum threshold required to conclude that the samples were perceived as distinct, at a 95% significance level. As expected, snacks enriched with microencapsulated extracts did not significantly affect sensory perception (*p* > 0.05).

Recent research has increasingly focused on the development of cookies enriched with functional ingredients derived from by-products of the food industry. Studies by [45,51,52,53] have investigated the incorporation of powdered citrus peel and pomace, common by-products of citrus processing, into shortbread cookie formulations. Sensory evaluations from these studies revealed notable differences between enriched cookies and control samples. Panelists reported perceivable changes in texture, color, and aroma/flavor. While the addition of citrus by-products often negatively impacted texture and taste, the enhanced citrus aroma was generally appreciated.

In contrast, our study employed an oatmeal-based cookie formulation enriched with functional extracts via microencapsulation, alongside the inclusion of chocolate—an approach modeled after traditional commercial health cookies. This combination appeared to mask perceptible differences in sensory attributes effectively. In fact, panelists were unable to distinguish between the control and the enriched versions during sensory evaluation. In addition, in recent research the results obtained with polyphenols encapsulation and incorporation into bakery products (bread and cookies) [54,55] confirm our results. Polyphenol encapsulation has proven to be an effective strategy for preserving antioxidant potential and structural integrity during high-temperature processing without compromising the technological quality or sensory acceptability of bakery products. These findings suggest promising opportunities for expanding the range of baked goods with new, functionally enriched formulations. Such diversification not only benefits consumers by offering more variety and personalized options but also provides strategic advantages to food manufacturers. Enhanced product differentiation, along with the ability to respond to evolving market demands, is a key driver for innovation in this sector.

## 4. Conclusions

This study demonstrated the feasibility of using citrus by-products, specifically lemon and orange peels, as a valuable source of phenolic compounds for the development of innovative functional foods. Through optimized hydroethanolic extraction and microencapsulation, flavonoid-rich extracts with high bioactive potential were successfully obtained. Major compounds identified included hesperidin, eriocitrin, and nobiletin. To address the limitations associated with polyphenol stability and sensory acceptability, microencapsulation was performed using a sodium alginate and corn starch blend (1% + 1.36%) and beads were produced with a 750 µm nozzle. This formulation provided high structural integrity, effective retention of flavonoids, and strong thermal resistance. After baking, the flavonoid content in enriched cookies remained high, meeting approximately one-third of the recommended daily intake. Therefore, the microencapsulation process ensured strong thermal resistance and preserved the functional properties of flavonoids during baking.

Sensory analysis using triangle tests revealed no significant differences (*p* > 0.05) between enriched and control cookies, suggesting that the inclusion of microencapsulated citrus extracts did not produce perceivable differences under the test conditions. This supports the suitability of the cookie matrix and the effectiveness of microencapsulation in masking undesirable sensory characteristics. By preserving the functional properties of flavonoids throughout processing, this study highlights the potential of microencapsulation as a strategy for incorporating bioactive compounds into bakery products. The approach demonstrates technical feasibility and aligns with sustainability goals by valorizing citrus residues. It offers a clean-label, health-oriented solution that meets the expectations of both consumers and the environment.

Nevertheless, some limitations should be acknowledged. This study did not include functional assays such as bioactivities, in vitro food digestion, or other cell-based models to confirm the biological activity of the encapsulated flavonoids-rich extracts and/or functional cookies. Future research should therefore focus on evaluating the bioaccessibility and bioavailability of these products, as well as their potential health benefits in conditions related to oxidative stress and inflammation. In addition, while the feasibility of microencapsulation was demonstrated at the laboratory scale, the economic viability for low-cost products such as cookies remains uncertain. A detailed techno-economic assessment, considering solvent recovery, energy efficiency, and process scaling, will be crucial for broader implementation within the food sector.

This work exemplifies the principles of a circular economy by transforming citrus processing waste into a functional food ingredient with nutritional relevance and market potential.

## Figures and Tables

**Table 1:** Microencapsulation operational settings.

Nozzle (μm)	Air Pressure (mbar)	Optimal Frequency Range (Hz)	Electrode (V)	Size Range of Produced Beads (μm)
80	500–1000	1300–3000	1000–2500	120–200
120	500–1000	950–2500	1000–2500	200–300
150	400–700	800–1800	1000–2500	260–350
200	400–700	600–1200	1000–2500	350–450
300	250–550	400–1000	1000–2500	550–700
450	250–550	200–900	1000–2500	700–1150
750	200–500	40–800	1000–2500	1150–1800
1000	200–500	40–650	1000–2500	1600–2400

**Table 2:** Key assumptions used to assess the production process costs of 450 mL of flavonoid-rich extract from orange or lemon by-products and 27 g dried microbeads.

Parameter	Flavonoid-Rich Extract Production		Microbeads Production	
	Price	Quantity	Price (€/kW)	Quantity
Energy	0.11 €/kW ^1^	1.67 kW	0.17 €/kW ^2^	12.11 kW
Citrus raw material	Not considered	300.00 g	-	-
Ethanol	14.00 €/kg	1.62 kg	-	-
Ultrapure water	0.51 €/kg	1.08 kg	-	-
Alginate	-	-	120.00 €/kg	9.00 g
Calcium chloride	-	-	1.60 €/kg	12.49 g
Overheads	80% of chemicals and energy costs			

^1^ Eurostat, statistics on electricity price for non-household consumers in Portugal [33]. ^2^ Eurostat, statistics on electricity price for non-household consumers in Italy [33].

**Table 3:** Formulation (%) of functional cookies enriched with orange and lemon polyphenol extracts and control cookies (without extract).

	Control Cookies (CC)	Orange Polyphenols Cookies (OPCs)	Lemon Polyphenols Cookies (LPCs)
Buckwheat flour	28.21	22.49	22.49
Oat flour and flakes	21.16	15.44	15.44
Cane sugar	16.93	16.93	16.93
Water	18.34	18.34	18.34
Corn seed oil	13.40	13.40	13.40
Chocolate chips	1.41	1.41	1.41
Baking powder	0.56	0.56	0.56
Dried beads with citrus extract	-	11.43	11.43

**Table 4:** Phenolic content (mg/L; n = 3) by HPLC-DAD of orange and lemon flavonoids-rich extracts.

Compounds	Orange Extract	Lemon Extract
Hesperidin	454.00 ± 16.00	100.98 ± 2.00
Narirutin	189.25 ± 7.01	-
Neoeriocitrin	223.88 ± 12.16	47.13 ± 2.71
Eriocitrin	62.40 ± 2.73	357.30 ± 2.45
Chlorogenic Acid	3.06 ± 0.36	-
Caffeic Acid	10.29 ± 0.75	-
Sinensetin	24.21 ± 0.77	-
Nobiletin	235.57 ± 6.19	-
Luteolin-7-O-glucoside	-	30.57 ± 0.27
Apigenin-7-O-glucoside	-	20.79 ± 0.35
Total Quantified (mg/L)	1206.66 ± 45.97	556.77 ± 7.78

**Table 5:** Total flavonoid content (TFC) of orange and lemon flavonoid extracts (expressed in mg quercetin eq./L).

	Orange Extract	Lemon Extract
Total flavonoid content (mg/L)	1960.1 ± 123.33	845.7 ± 58.61

**Table 6:** Total flavonoid content (mg quercetin eq. kg^−1^) of oven cooking tests at 180 °C and 230 °C.

	Lemon Extract	Lemon Dried Microbeads	Orange Extract	Orange Dried Microbeads
Before oven test	845.7 ± 58.61 ^a^	12,047.25 ± 1036.87 ^a^	1960.1 ± 123.33 ^a^	11,917.11 ± 996.60 ^a^
After 30′ 180 °C	448.10 ± 23.79 ^b^	10,398.70 ± 800.25 ^a^	1034.38 ± 113.82 ^b^	10,289.09 ± 817.38 ^ab^
After 30′ 230 °C	-	11,234.37 ± 1073.24 ^a^	-	10,094.70 ± 720.53 ^b^
*p*-value	0.0004	0.1996	0.0007	0.0744

Notes: values are presented as mean ± SD (n = 3). Differences between samples were analyzed using one-way ANOVA followed by Tukey’s multiple range test. Within each column, values sharing different superscript letters (a,b) are significantly different at *p*-values shown in the Table.

**Table 7:** Laboratory-scale estimation of production costs for flavonoid-rich extracts and microbeads (€/kg product).

	Flavonoids Extract Production (€/kg Product)	Microbeads Production (€/kg Product)
Energy		
Stirrer + Centrifuge + Reverse Osmosis	0.19	-
Buchii encapsulator + drying incubator	-	2.01
Chemicals		
Ethanol	22.68	-
Ultrapure water	0.55	-
Alginate	-	1.08
Calcium chloride	-	0.02
Overheads	18.72	2.49
Total cost per stage (€)	42.16	5.60
Total cost for production (€)	47.73	

**Table 8:** Flavonoids enriched-cookies analysis (mg quercetin eq./100 g).

	Control Cookies (CCs)	Orange Polyphenols Cookies (OPCs)	Lemon Polyphenols Cookies (LPCs)
Total flavonoids (mg/100 g)	-	166.11 ± 37.36	177.13 ± 24.99

## Data Availability

The data presented in this study are available on request from the corresponding author due to privacy.

## References

[1] Vilas-boas A.A., Magalhães D., Campos D.A., Porretta S., Dellapina G., Poli G., Istanbullu Y., Demir S., Mart Á., Mart S. (2022). Innovative Processing Technologies to Develop a New Segment of Functional Citrus-Based Beverages: Current and Future Trends. Foods.

[2] Magalhães D., Vilas-Boas A.A., Teixeira P., Pintado M. (2023). Functional Ingredients and Additives from Lemon By-Products and Their Applications in Food Preservation: A Review. Foods.

[3] Visvanathan R., Williamson G. (2022). Review of Factors Affecting Citrus Polyphenol Bioavailability and Their Importance in Designing in Vitro, Animal, and Intervention Studies. Compr. Rev. Food Sci. Food Saf..

[4] Pieracci Y., Pistelli L., Cecchi M., Pistelli L., De Leo M. (2022). Phytochemical Characterization of Citrus-Based Products Supporting Their Antioxidant Effect and Sensory Quality. Foods.

[5] Anticona M., Blesa J., Frigola A., Esteve M.J. (2020). High Biological Value Compounds Extraction from Citruswaste with Non-Conventional Methods. Foods.

[6] Putnik P., Bursać Kovačević D., Režek Jambrak A., Barba F.J., Cravotto G., Binello A., Lorenzo J.M., Shpigelman A. (2017). Innovative “Green” and Novel Strategies for the Extraction of Bioactive Added Value Compounds from Citruswastes—A Review. Molecules.

[7] Cory H., Passarelli S., Szeto J., Tamez M., Mattei J. (2018). The Role of Polyphenols in Human Health and Food Systems: A Mini-Review. Front. Nutr..

[8] Williamson G., Holst B. (2008). Dietary Reference Intake (DRI) Value for Dietary Polyphenols: Are We Heading in the Right Direction?. Br. J. Nutr..

[9] Bjelakovic G., Nikolova D., Simonetti R.G., Gluud C. (2004). Antioxidant Supplements for Prevention of Gastrointestinal Cancers: A Systematic Review and Meta-Analysis. Lancet.

[10] Osakabe N., Shimizu T., Fujii Y., Fushimi T., Calabrese V. (2024). Sensory Nutrition and Bitterness and Astringency of Polyphenols. Biomolecules.

[11] Dabas D. (2018). Polyphenols as Colorants. Adv. Food Technol. Nutr. Sci. Open J..

[12] De Rossi L., Rocchetti G., Lucini L., Rebecchi A. (2025). Antimicrobial Potential of Polyphenols: Mechanisms of Action and Microbial Responses—A Narrative Review. Antioxidants.

[13] Speisky H., Shahidi F., Costa de Camargo A., Fuentes J. (2022). Revisiting the Oxidation of Flavonoids: Loss, Conservation or Enhancement of Their Antioxidant Properties. Antioxidants.

[14] Szente L., Sohajda T., Fenyvesi É. (2021). Encapsulation for Masking Off-Flavor and Off-Tasting in Food Production. Functionality of Cyclodextrins in Encapsulation for Food Applications.

[15] Behrens M. (2024). The Growing Complexity of Human Bitter Taste Perception. J. Agric. Food Chem..

[16] Lesschaeve I., Noble A.C. (2005). Polyphenols: Factors Influencing Their Sensory Properties and Their Effects on Food and Beverage Preferences. Am. J. Clin. Nutr..

[17] Ramos-Hernández J.A., Cruz-Salas C.N., González-Gutiérrez K.N., González-Cruz E.M., Prieto López C. (2023). Encapsulation of Plant Secondary Metabolites of Industrial Interest. Advances in Plant Biotechnology.

[18] Tekin İ., Özcan K., Ersus S. (2023). Optimization of Ionic Gelling Encapsulation of Red Beet (*Beta Vulgaris* L.) Juice Concentrate and Stability of Betalains. Biocatal. Agric. Biotechnol..

[19] Flamminii F., Paciulli M., Di Michele A., Littardi P., Carini E., Chiavaro E., Pittia P., Di Mattia C.D. (2021). Alginate-Based Microparticles Structured with Different Biopolymers and Enriched with a Phenolic-Rich Olive Leaves Extract: A Physico-Chemical Characterization. Curr. Res. Food Sci..

[20] Kurozawa L.E., Hubinger M.D. (2017). Hydrophilic Food Compounds Encapsulation by Ionic Gelation. Curr. Opin. Food Sci..

[21] Castro N., Durrieu V., Raynaud C., Rouilly A., Rigal L., Quellet C. (2016). Melt Extrusion Encapsulation of Flavors: A Review. Polym. Rev..

[22] Román S., Sánchez-Siles L.M., Siegrist M. (2017). The Importance of Food Naturalness for Consumers: Results of a Systematic Review. Trends Food Sci. Technol..

[23] Dias R., Oliveira H., Fernandes I., Simal-Gandara J., Perez-Gregorio R. (2021). Recent Advances in Extracting Phenolic Compounds from Food and Their Use in Disease Prevention and as Cosmetics. Crit. Rev. Food Sci. Nutr..

[24] European Parliament Council of the European Union (2008). Regulation (EC) No 1333/2008 of the European Parliament and of the Council of 16 December 2008 on Food Additives.

[25] Vilas-boas A.A., Campos D.A., Nunes C., Ribeiro S. (2020). Polyphenol Extraction by Different Techniques for Valorisation of Non-Compliant Portuguese Sweet Cherries towards a Novel Antioxidant Extract. Sustainability.

[26] Zhishen J., Mengcheng T., Jianming W. (1999). The Determination of Flavonoid Contents in Mulberry and Their Scavenging Effects on Superoxide Radicals. Food Chem..

[27] Iswandana R., Amangkoe E., Isnaini R. (2018). Tetrandrine Beads Using Alginate/Polyvinyl Alcohol and Alginate-Carboxymethyl Cellulose: Not Ideal as Colon-Targeted Dosage Form. J. Pharm. Negat. Results.

[28] López-Córdoba A., Deladino L., Martino M. (2014). Corn Starch-Calcium Alginate Matrices for the Simultaneous Carrying of Zinc and Yerba Mate Antioxidants. LWT—Food Sci. Technol..

[29] Kanokpanont S., Yamdech R., Aramwit P. (2018). Stability Enhancement of Mulberry-Extracted Anthocyanin Using Alginate/Chitosan Microencapsulation for Food Supplement Application. Artif. Cells Nanomed. Biotechnol..

[30] Macías-Cortés E., Gallegos-Infante J.A., Rocha-Guzmán N.E., Moreno-Jiménez M.R., Medina-Torres L., González-Laredo R.F. (2019). Microencapsulation of Phenolic Compounds: Technologies and Novel Polymers. Rev. Mex. Ing. Quim..

[31] Gao Y., Xia W., Shao P., Wu W., Chen H., Fang X., Mu H., Xiao J., Gao H. (2022). Impact of Thermal Processing on Dietary Flavonoids. Curr. Opin. Food Sci..

[32] Green D.W., Southard M.Z. (2019). Perry’s Chemical Engineers’ Handbook.

[33] Eurostat (2025). Electricity Prices for Non-Household Consumers—Bi-Annual Data (from 2007 Onwards).

[34] (2021). Sensory Analysis-Methodology-Triangle Test.

[35] (2014). Guida Generale per La Progettazione Di Locali Di Prova.

[36] M’hiri N., Ioannou I., Ghoul M., Boudhrioua N.M. (2014). Extraction Methods of Citrus Peel Phenolic Compounds. Food Rev. Int..

[37] M’hiri N., Ioannou I., Ghoul M., Mihoubi Boudhrioua N. (2017). Phytochemical Characteristics of Citrus Peel and Effect of Conventional and Nonconventional Processing on Phenolic Compounds: A Review. Food Rev. Int..

[38] Bağdatlı İ., Khalily R. (2022). Effects of Different Solvent Extractions on the Total Phenolic Content and Antioxidant Activity of Lemon and Orange Peels. Eurasian J. Food Sci. Technol..

[39] Özcan M.M., Ghafoor K., Al Juhaimi F., Uslu N., Babiker E.E., Mohamed Ahmed I.A., Almusallam I.A. (2021). Influence of Drying Techniques on Bioactive Properties, Phenolic Compounds and Fatty Acid Compositions of Dried Lemon and Orange Peel Powders. J. Food Sci. Technol..

[40] Singh B., Singh J.P., Kaur A., Singh N. (2020). Phenolic Composition, Antioxidant Potential and Health Benefits of Citrus Peel. Food Res. Int..

[41] Andrade M.A., Barbosa C.H., Shah M.A., Ahmad N., Vilarinho F., Khwaldia K., Silva A.S., Ramos F. (2022). Citrus By-Products: Valuable Source of Bioactive Compounds for Food Applications. Antioxidants.

[42] Mateus A.R.S., Mariño-Cortegoso S., Barros S.C., Sendón R., Barbosa L., Pena A., Sanches-Silva A. (2024). Citrus By-Products: A Dual Assessment of Antioxidant Properties and Food Contaminants towards Circular Economy. Innov. Food Sci. Emerg. Technol..

[43] Nanditha B., Prabhasankar P. (2008). Antioxidants in Bakery Products: A Review. Crit. Rev. Food Sci. Nutr..

[44] Melini V., Melini F., Luziatelli F., Ruzzi M. (2020). Functional Ingredients from Agri-Food Waste: Effect of Inclusion Thereof on Phenolic Compound Content and Bioaccessibility in Bakery Products. Antioxidants.

[45] Imeneo V., Romeo R., Gattuso A., De Bruno A., Piscopo A. (2021). Functionalized Biscuits with Bioactive Ingredients Obtained by Citrus Lemon Pomace. Foods.

[46] de Castro L.A., Lizi J.M., das Chagas E.G.L., Carvalho R.A.d., Vanin F.M. (2020). From Orange Juice By-Product in the Food Industry to a Functional Ingredient: Application in the Circular Economy. Foods.

[47] Aguirre-Calvo T.R., Aguirre-Calvo D., Perullini M., Santagapita P.R. (2021). A Detailed Microstructural and Multiple Responses Analysis through Blocking Design to Produce Ca(II)-Alginate Beads Loaded with Bioactive Compounds Extracted from by-Products. Food Hydrocoll. Health.

[48] Restrepo-Serna D.L., Cardona-Alzate C.A. (2024). Pre-Feasibility Assessment to Obtain an Extract Rich in Hesperidin from Orange Peel: A Comparison of Extraction Technologies Conventional and Non-Conventional. Sustain. Chem. Pharm..

[49] Ortiz-Sanchez M., Solarte-Toro J.C., Orrego-Alzate C.E., Acosta-Medina C.D., Cardona-Alzate C.A. (2021). Integral Use of Orange Peel Waste through the Biorefinery Concept: An Experimental, Technical, Energy, and Economic Assessment. Biomass Convers. Biorefin..

[50] Adeyi O., Oke E.O., Okolo B.I., Adeyi A.J., Otolorin J.A., Nwosu-Obieogu K., Adeyanju J.A., Dzarma G.W., Okhale S., Ogu D. (2022). Process Optimization, Scale-up Studies, Economic Analysis and Risk Assessment of Phenolic Rich Bioactive Extracts Production from Carica Papaya L. Leaves via Heat-Assisted Extraction Technology. Heliyon.

[51] Abreu J., Quintino I., Pascoal G., Postingher B., Cadena R., Teodoro A. (2019). Antioxidant Capacity, Phenolic Compound Content and Sensory Properties of Cookies Produced from Organic Grape Peel (*Vitis Labrusca*) Flour. Int. J. Food Sci. Technol..

[52] Al-Saab A.H., Gadallah M.G.E. (2021). Phytochemicals, Antioxidant Activity and Quality Properties of Fibre Enriched Cookies Incorporated with Orange Peel Powder. Food Res..

[53] Kausar T., Saeed E., Hussain A., Firdous N., Bibi B., Kabir K., Ul An Q., Ali M.Q., Najam A., Ahmed A. (2024). Development and Quality Evaluation of Cookies Enriched with Various Levels of Grapefruit Pomace Powder. Discov. Food.

[54] Chernenko S. (2025). Encapsulation of Polyphenols in Baked Goods: A Strategy for Enhancing Stability and Antioxidant Activity. Technol. Audit. Prod. Reserves.

[55] Kaderides K., Mourtzinos I., Goula A.M. (2020). Stability of Pomegranate Peel Polyphenols Encapsulated in Orange Juice Industry By-Product and Their Incorporation in Cookies. Food Chem..

